# Evaluation of the usefulness of EQ-5D as a patient-reported outcome measure using the Paretian classification of health change among patients with chronic heart failure

**DOI:** 10.1186/s41687-020-00216-7

**Published:** 2020-06-26

**Authors:** Åsa Jonsson, Lotti Orwelius, Ulf Dahlstrom, Margareta Kristenson

**Affiliations:** 1 Department of Medicine, Division of Cardiology, County Hospital Ryhov, 551 85 Jönköping, Sweden; 2 Department of Intensive Care, Biomedical and Clinical Sciences, Linköping University, County Council of Östergötland, Linköping, Sweden; 3 Department of Cardiology and Department of Health, Medicine and Caring Sciences, Linköping University, Linköping, Sweden; 4 Department of Public Health Sciences and Department of Health, Medicine and Caring Sciences, Linköping University, Linköping, Sweden

**Keywords:** Health-related quality of life; co-morbidity, Heart failure, EQ-5D, Paretian classification of health change, Registry, Quality registries

## Abstract

**Purpose:**

The aim of this study was to evaluate the usefulness of EQ-5D as a patient-reported outcome measure using different analytical methods. Especially we used the Paretian Classification of Health Change, to see if this gave better information compared to measures that are more traditional. For the evaluation we used data from patients with chronic heart failure (HF).

**Methods:**

We compared results of EQ-5D at baseline and at 1 year’s follow up for HF patients with preserved or reduced ejection fraction (EF), HFpEF (EF > 50%, *n* = 930) and HFrEF (EF < 40%, *n* = 3831) using individual patient data from the Swedish Heart Failure Registry. Statistical analysis included EQ-5D index and proportions for all five dimensions of the EQ-5D. In addition, we also used the Paretian classification of Health Change to judge overall improvements (improved in at least one dimension and not worsened in any other dimension) or worsening (vice versa) in EQ-5D profiles.

**Results:**

Mean EQ-5D index showed minor changes at the one-year follow-up, likewise in both groups. The proportions reporting moderate, or severe, problems increased for all five dimensions of the EQ-5D in the HFpEF group. In the HFrEF group this was seen only for three dimensions, with no change for “anxiety/depression” and reduction of problems for “usual activities “. The Paretian classification showed that 24% (*n* = 200) of the HFpEF group and 34% (*n* = 1059) of the HFrEF group reported overall improvement while 43% (*n* = 355) and 39% (*n* = 1212) respectively reported overall worsening. Multiple logistic regressions showed different patterns of determinants e.g. that treatment in a cardiology clinic only affected overall health outcome in the HFrEF group.

**Conclusion:**

The usefulness of EQ-5D is dependent on the analytical method used. While the index showed minor differences between groups, analyses of specific dimensions showed different patterns of change in the two groups with better prognosis for the HFrEF group. The Paretian classification of Health Change could further identify subgroups that showed overall improvements or overall worsening. This method can therefore help to identify needs for more tailored interventions in health services.

## Introduction

In recent decades, it has been increasingly recognised that outcome measures within health services need to include beyond clinical observations, laboratory results and other examinations, also patients’ own assessments of their outcomes. Therefore patient-reported outcome measures (PROM) are increasingly in demand. PROM instruments can be disease-specific, or generic. The latter focus on outcomes in terms of general health and health-related quality of life (HRQoL) including physical, mental, and social well-being, and functional ability. This is particularly important for patients with chronic diseases for whom the objective of the health service is to limit the effects and symptoms of the disease as much as possible and to help the patients to live with the disease with best possible self-rated health and HRQoL. Measures of HRQoL are also independent predictors of prognosis in terms of both morbidity and mortality [1, 2]. HRQoL is therefore an important outcome measure in clinical practice. To be useful clinically, PROM measures in terms of HRQoL must be easy for patients to use and also provide useful information about important problems to help staff to focus on the patient’s main concerns. For this, several instruments have been developed [1].

The most commonly used generic instruments are the Short Form-36 (SF-36) [3] and EQ-5D [4]. These were developed for different purposes, the SF-36 being aimed to illustrate a broad health profile, and the EQ-5D a measure developed for the creation of a single composite measure (index) for health economic analyses [5, 6]. Over time both have broadened their uses, so that today SF-36 has indexes that can be used in health economic analyses, and subsequently, the interest to use each dimension of EQ-5D is rising. The EQ-5D instrument is brief and simple for patients to complete and places a minimal burden on the respondent, and is currently used in many Swedish National Quality Registries [1]. However, so far, the most common way to use the EQ-5D is to look at the index value, not the specific dimensions. There is also a need to identify models of how to analyse and interpret results to guide improvement [1].

Heart Failure (HF) is one of the major chronic diseases and is a complex clinical syndrome that can result from any cardiac structural or functional disorder that impairs the ability of the ventricle to fill or eject blood. The most common clinical signs of HF are dyspnoea, fatigue, and peripheral oedema. The symptoms can vary from patient to patient and are not specific to HF, so the diagnosis can be difficult to verify particularly in the early stages [7, 8]. Affected patients are commonly classified according to left ventricular ejection fraction (EF) into those who have a preserved ejection fraction (HFpEF, EF > 50%) or those in whom it is reduced (HFrEF, EF < 40%). The category HF with mid-range EF (HFmrEF); EF40–49%)) included in the recent guidelines resembles regarding characteristics and outcomes patients with HFrEF [9]. All patients with HF have a significant morbidity and mortality but in contrast to the efficacy of evidence-based treatment in patients with HFrEF, so far no treatment has been clearly shown to improve the outcomes in HFpEF or HFmrEF [9]. Apart from having a poor prognosis in terms of premature mortality, [10] HF is a disabling disease that limits physical, social, and mental well-being, and leads to reduced HRQoL. Affected patients have been shown to have reduced HRQoL, both when compared to a normal population [11, 12] and to patients with other chronic diseases such as angina pectoris, atrial fibrillation, and chronic obstructive pulmonary disease [11, 13].

Despite the fact that HRQoL is an important measure in the management of patients, instruments to measure this are rarely used in clinical practice. Previously, the main use of PROM has been in clinical trials, but they are now being used more often in the Swedish National Quality Registries [1]. One of these is the Swedish HF Registry (SwedeHF). In this study, we have therefore used data from the patients with HF in the SwedeHF registry as an example for our purposes.

Our aim was to evaluate the usefulness of EQ-5D as a PROM by using different analytical methods. In addition to the traditional EQ-5D index we also analysed specific dimensions and, in particular, we used the Paretian Classification of Health Change [14] based on these dimensions. Data from SwedeHF, at baseline and at 1-year follow up were used, and changes in EQ-5D for patients with HFpEF and HFrEF were compared.

## Methods

The protocol of this prospective study, which used data from SwedeHF, has been described previously [15]. In brief, we included patients with clinically diagnosed HF. Initial recording of patients’ information on this web-based database was done at discharge from hospital or at an outpatient visit and includes a broad range of information, including demography, coexisting diseases, diagnostic investigations and results, medication and HRQoL (EQ-5D). One year after the initial recording, a new questionnaire was sent to all patients to collect information about current medication, functional capacity, and HRQoL. Individual patients were not required to give consent, but they were informed of entry into national registries and allowed to opt out according to the Swedish data legislation. The protocol, record form, and annual reports are available at www.ucr.uu.se/rikssvikt-en/. Establishment of the registry and analysis of data were approved by a multisite ethics committee and conform to the requirements of the Declaration of Helsinki.

**Fig. 1:**
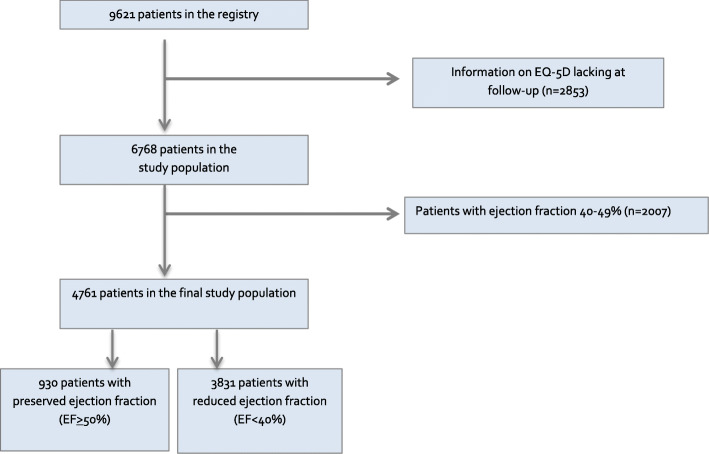
Flow chart over the selection process of the patients from the Swedish HF Registry (SwedeHF)

Data from the EQ-5D questionnaire were included in the registry from February 1, 2008. From this day until November 11, 2013 there were 9621 unique index recordings with baseline EQ-5D data from 65 out of 75 hospitals in Sweden. The index date was defined as the date when the first record was entered. Patients who had no EQ-5D at the one-year follow up were excluded (*n* = 2853) (Fig. 1). For this study, patients with HFpEF (*n* = 930), and HFrEF (*n* = 3831) were included, which left a total of 4761 patients in the study population (Fig. 1).

The EQ-5D instrument was developed by an international multidisciplinary research group and consists of two parts, a health state classification scheme of five items (EQ-5D self-classifier) and a visual analogue scale (EQ-VAS) [5, 6]. In this study we have used only data from the EQ-5D self-classifier, which includes five dimensions (mobility, self-care, daily activities, pain/discomfort, and anxiety/depression). In the version that we used (EQ-5D-3L) each question has a choice of three responses (1 = no problems, 2 = moderate problems, and 3 = severe problems). The five dimensions have most commonly been used as a basis for an index to define a state of health, built on a combination of answers of the five dimensions (3^5^ = 243 theoretically possible states of health). The upper value of the index is 1, corresponding to full health according to EQ-5D, the value 0 corresponds to a state as bad as death, and states valued as being worse than death have negative index value. It is also possible to use information from each dimension and this was done in this study.

In addition, the following characteristics were retrieved from the SwedeHF register; sex, age, smoking habits, clinic attended for care (cardiology vs. medicine or geriatrics), follow up referral to hospital vs. primary care, follow up referral to out-patient HF nurse and New York Heart Association (NYHA) classification (class I and II are equivalent to mild to moderately severe HF) [16]. We also recorded symptoms; fatigue and shortness of breath, medical history; duration of heart failure, ischaemic heart disease, previous myocardial infarction, hypertension, atrial fibrillation/flutter, diabetes, pulmonary disease and coexisting conditions, heart rate, laboratory results; haemoglobin, renal function (estimated glomerular filtration rate (e-GFR)) and medication; drugs documented were Angiotensin Converting Enzyme Inhibitor (ACEI)/Angiotensin II Receptor Blocker (ARB), ß-blocker, Mineralocorticoid Receptor Antagonists (MRA), and Diuretic. Normal renal function was defined as an e-GFR of more than 60 ml/min/1.73m^2^ [17]. Anaemia was defined as haemoglobin concentration of less than 130 g/L in men and less than 120 g/L in women.

### Statistical analyses

Descriptive statistics have been presented as percent (%) and number (n). For comparison of proportions (categorical variables) the Chi-square test has been used. Analyses were thereafter done step by step as follows:

- Calculation of EQ-5D index, and confidence intervals, for each participant and mean levels for the two groups. Thereafter comparison of changes in mean level of EQ-5D index between baseline and follow up separately for groups of patients with HFpEF and HFmrEF using Paired *t-*test. The value set to calculate the EQ-5D index is based on data from a British study used in many countries who like Sweden do not have their own reference data [18].
- Calculation of proportions of patients who reported at least some problems (i.e. at level 2 or 3; moderate, or severe problems) for each dimension of EQ-5D. Thereafter comparison of changes of these proportions between baseline and follow up separately for groups of patients with HFpEF and HFrEF using the Wilcoxon Signed Ranks Test.
- Using the Paretian Classification of Health Change, the overall change in EQ-5D levels were calculated for the groups of patients with HFpEF and HFrEF. This was done in terms of percentages improved, worsened, mixed changes, no problems or no change. “Improvement” was defined for each participant as improvement in at least one dimension and no worsening in any other dimension. Correspondingly, “worsening” was defined as worsening in at least one dimension, and no improvement in any other dimension. When there was a mix of “better” and “worse” across dimensions, the group was named “mixed”. If there were no problems in any dimension, both at baseline and follow up, the group was named “no problems” and if no changes at all, the group was named “no change”.

Since the group “no problems” is a subgroup of the” no change group”, the analyses of Paretian classification were made in three steps. In the first step, the group “no problems,”, was included in the study population. In the second step, this group was defined as a discrete group (“no problem”), and in the third step the group “no problems” was excluded from analysis.

d) Finally, stepwise multiple logistic regression analyses were performed, for each of the patient groups HFpEF and HFrEF separately, to identify the variables at baseline that significantly predicted overall improvement and overall worsening. In all four models the reference group was all other patients except the group “no problems”.

Variables examined in the models were; sex, age, smoking habits, clinic attended for care (cardiology vs. medicine or geriatrics), follow up referral to hospital vs. primary care, follow up referral to outpatient HF nurse, NYHA classification, fatigue, shortness of breath, duration of heart failure > 6 months, ischaemic heart disease, previous myocardial infarction, hypertension, atrial fibrillation/flutter, diabetes, pulmonary disease, anaemia, mean heart rate, reduced renal function (e-GFR < 60 μmol/L), and drugs taken: ACEI/ARB, ß-blocker, MRA, and Diuretic.

Statistical analyses were made with the aid of IBM SPSS Statistics (version 22, IBM Corp, Armonk, NY, USA). All tests were two-tailed, and the level of significance was defined as a *p*-value < 0.05.

## Results

Patients with HFpEF were (compared to patients with HFrEF) more often female, over 75 years old, had at baseline more signs of multiple disease, a longer duration of their HF and more often a history of hypertension, atrial fibrillation/flutter, diabetes, pulmonary disease, anaemia, multiple co-morbidity and reduced e-GFR (Table 1). Patients with HFrEF were more often in NYHA class III or IV. They had, at baseline, more often a history of ischaemic heart disease (IHD), previous myocardial infarction and were more often on treatment with HF specific medication; ACEI:s or ARB:s, β-blocking agents and MRA:s (Table 1). A higher proportion of the HFrEF patients were followed up at hospital or/and had visits to a HF nurse.

**Table 1:** Baseline characteristics % (n) for the two study groups HFpEF (> 50%) and HFrEF (< 40%) *n* = 4761

Variables	HFpEF > 50% % (*n* = 930)	HFrEF < 40% % (*n* = 3831)	*p*-value
Demographic characteristics			
Female sex	49 (456)	27 (1047)	< 0.001
Age (over 75 years old)	60 (555)	36 (1363)	< 0.001
Smoker (*n* = 4290)	10 (80)	13 (469)	0.007
^a^ Cardiology clinic (*n* = 4419)	61 (452)	59 (2170)	0.362
^a^ Follow up referral to hospital (*n* = 4673)	61 (560)	87 (3275)	< 0.001
Follow up referral to out-patient heart failure nurse (*n* = 4657)	55 (503)	76 (2839)	< 0.001
Medical history			
NYHA classification grades III / IV (*n* = 4476)	33 (274)	38 (1374)	0.014
Fatigue - pronounced/severe limitations (*n* = 4726)	34 (314)	29 (1098)	0.002
Shortness of breath - pronounced/severe limitations (*n* = 4731)	35 (319)	31 (1167)	0.020
Duration of heart failure ≥6 months (*n* = 4723)	46 (427)	36 (1360)	< 0.001
Ischaemic heart disease (*n* = 4474)	36 (323)	44 (1580)	< 0.001
Previous myocardial infarction (*n* = 2850)	26 (126)	35 (820)	< 0.001
Hypertension (*n* = 4635)	61 (561)	44 (1646)	< 0.001
Atrial fibrillation/flutter (*n* = 4738)	55 (509)	43 (1624)	< 0.001
Diabetes (*n* = 4741)	25 (229)	20 (767)	0.002
Pulmonary disease (*n* = 4693)	19 (174)	14 (515)	< 0.001
Anaemia (*n* = 4760)	33 (306)	23 (881)	< 0.001
^a^Coexisting condition			
1 disease	14 (128)	21 (822)	< 0.001
2 diseases	25 (229)	24 (915)	
3 diseases	26 (245)	21 (795)	
4 diseases	31 (291)	24 (902)	
Mean heart rate ≥ 70 bpm (*n* = 4701)	53 (482)	55 (2085)	0.166
Laboratory examinations			
Reduced renal function (e-GFR < 60 μmol/L)	46 (431)	36 (1376)	< 0.001
Drugs being taken			
ACEI/ARB	82 (761)	96 (3684)	< 0.001
β-blocker (*n* = 4750)	82 (757)	93 (3559)	< 0.001
MRA (*n* = 4738)	21 (199)	31 (1185)	< 0.001
Diuretic (*n* = 4738)	77 (712)	75 (2848)	0.169

^a^ NOTE: Cardiology clinic; Cardiology vs. Medicine/Geriatrics. Follow up referral to hospital; Hospital vs. Primary care; Coexisting conditions; Ischaemic heart disease, Previous myocardial infarction, Hypertension, Atrial fibrillation/flutter, Pulmonary disease, Diabetes, Anaemia

*Abbreviations*: *ACEI* Angiotensin Converting Enzyme Inhibitor, *ARB* Angiotensin II Receptor Blocker, *e-GFR* estimated Glomerular Filtration Rate, *HFrEF* Heart failure with reduced ejection fraction, *HFpEF* Heart failure with preserved ejection fraction, *MRA* Mineralocorticoid Receptor Antagonist, *NYHA* New York Heart Association

**Fig. 2:**
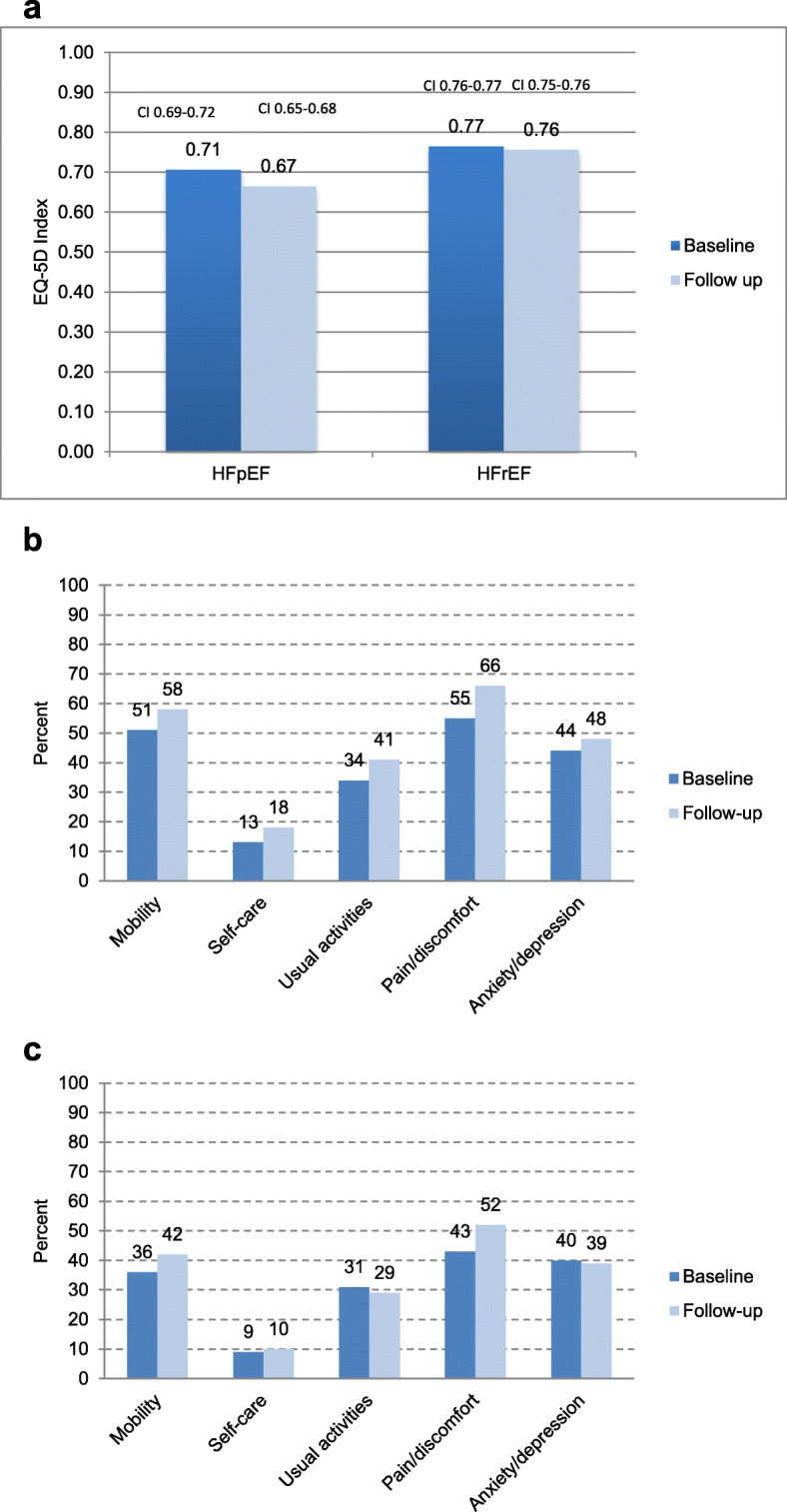
**a** Mean levels of EQ-5D index with confidence intervals in the two study groups, HFpEF (EF > 50%) and HFrEF (EF < 40%) at baseline and at follow up. **b** Proportions of patients with HFpEF (EF > 50%) reporting at least some problems (levels 2 or 3) for each dimension of EQ-5D at baseline and at follow up. **c** Proportions of patients with HFrEF (EF < 40%) reporting at least some problems (levels 2 or 3) for each dimension of EQ-5D, at baseline and at follow up

Figure 2, shows the comparisons between baseline and 1-year follow up for mean EQ-5D index and Fig. 2b and c proportions of patients with some problems (level 2 and 3; moderate/high problems) in each EQ-5D dimension in patients for the HFpEF and HFrEF groups separately.

For patients with HFpEF mean value of the EQ-5D index was lower at follow up 0.67 (CI 0.65–0.68) compared with baseline 0.71 (CI 0.69–0.72) (*p* < 0,001) with no difference over time in patients with HFrEF; 0.76 (CI 0.75–0.76) at follow up and 0.77 (CI 0.76–0.77) at baseline (*p* = 0,051). The patients in the HFpEF group showed higher proportions that reported some problems in all five dimensions at follow up (Table 2 and Fig. 2b). In the HFrEF group this was seen in three dimensions with no differences for Anxiety/Depression and lower proportions for Usual Activities (Table 2 and Fig. 2c).

**Table 2:** Prevalence (number and proportions) of the two groups of patients HFpEF and HFrEF reporting at least some problems (levels 2 or 3) for each dimension of EQ-5D at baseline and at follow up and changes between baseline and follow up

	Number of patients reporting problems levels 2 and 3						Change in numbers of patients reporting problems levels 2 and 3		Percent change in numbers of patients reporting problems levels 2 and 3	
	HFpEF > 50%,			HFrEF < 40%			EF > 50%,	EF < 40%	EF > 50%,	EF < 40%
	Baseline % (n)	Follow up % (n)	*p*-value	Baseline % (n)	Follow up % (n)	*p*-value				
Mobility	51 (473)	58 (540)	< 0.001	36 (1396)	42 (1598)	< 0.001	+ 67	+ 202	+ 14%	+ 14%
Self-care	13 (121)	18 (163)	0.001	9 (341)	10 (402)	0.003	+ 42	+ 61	+ 35%	+ 18%
Usual activities	34 (320)	41 (382)	< 0.001	31 (1203)	29 (1120)	0.013	+ 62	- 83	+ 19%	- 7%
Pain/discomfort	55 (516)	66 (618)	< 0.001	43 (1644)	52 (1990)	< 0.001	+ 102	+ 346	+ 20%	+ 21%
Anxiety/depression	44 (406)	48 (444)	0.025	40 (1525)	39 (1505)	0.550	+ 38	- 20	+ 9,4%	- 1%
EQ-5D Index (mean levels)	0.71	0.67	< 0.001	0.77	0.76	0.051				

**Fig. 3:**
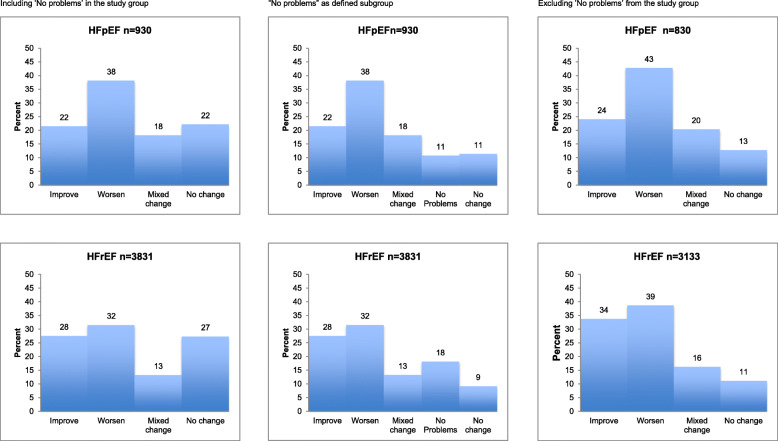
Proportions of patients in the two groups HFpEF (EF > 50%) and HFrEF (EF < 40%) in different groups of Paretian Classification of Health Change as measured by the EQ-5D

Figure 3 shows the distribution of changes according to the Paretian classification of Health Change for the two subgroups of HF in three models. In the third model, excluding the group with “no problems”, among the patients in the HFpEF group 24% (*n* = 200) showed overall improvement, 43% (*n* = 355) showed overall worsening, 20% (*n* = 169) showed mixed change and 13% (*n* = 106) showed no change The corresponding figures for the patients in the HFrEF group are 34% (*n* = 1059) improvement, 39% (*n* = 1212) worsening, 16% (*n* = 510) mixed change and 11% (*n* = 352) no change. The proportion with no problems was smaller in the HFpEF group 11% (*n* = 102) than the HFrEF group 18% (*n* = 690). However, the same pattern of a lower proportion with overall improvement and of higher proportion of overall worsening in the HFpEF group, compared with the HFrEF group, was seen independently of including or excluding the group “no problems” in the analyses.

In multiple logistic regression analysis within the HFpEF group, factors at baseline, related to overall improvement were severe problems with shortness of breath and good renal function. Determinants for overall worsening were atrial fibrillation, low use of diuretics, and reduced renal function at baseline.

Factors, at baseline, affecting overall improvement within the HFrEF group were being treated at a cardiology clinic, good renal function, high fatigue, low experience of IHD and pulmonary disease. Factors significantly affecting overall worsening were male gender, not being treated at a cardiology clinic, low fatigue, low shortness of breath, prevalent pulmonary disease and low use of MRA (Tables 3 and 4).

**Table 3:** Results from multiple logistic regression analysis with Odds Ratios (OR) of factors affecting improved outcomes in the two groups HFpEF and HFrEF

	Improved			
	HFpEF ≥50%		HFrEF < 40%	
	OR	*p*-value	OR	*p*-value
Clinic (cardiology vs. medicine/geriatric clinic)			1.428	< 0.001
Fatigue (pronounced/severe limitations vs. light)			1.588	< 0.001
Shortness of breath (pronounced/severe limitations vs. light)	1.596	0.005		
Ischaemic heart disease (yes/no)			0.787	0.004
Pulmonary disease (yes/no)			0.742	0.014
Reduced renal function (yes/no)	0.709	0.037	0.712	< 0.001

All analyses controlled for: Sex (female vs. male), Age (over 75 years old), Smoker (yes/no), Clinic (Cardiology vs. Medicine/Geriatrics), Follow up referral to hospital (Hospital vs. Primary care) Follow up referral to out-patient HF nurse (yes/no), NYHA classification grades III/IV vs. 1/II, Fatigue – pronounced/severe limitations, Shortness of breath – pronounced/severe limitations, Duration of heart failure > 6 months), Ischaemic heart disease, Previous myocardial infarction, Hypertension, Atrial fibrillation/flutter, Diabetes, Pulmonary disease, Anaemia, Mean heart rate bpm > 70, Renal function (e-GFR < 60 μmol/L), ACEI/ARB, ß-blocker, MRA, Diuretic

*Abbreviations*: *ACEI* angiotensin converting enzyme inhibitor, *ARB* angiotensin II receptor blocker, *EF* ejection fraction, *e-GFR* estimated glomerular filtration, *HFrEF* Heart Failure with Reduced Ejection Fraction, *HFpEF* Heart Failure with Preserved Ejection Fraction, *MRA* mineralocorticoid receptor antagonist, *NYHA* New York Heart Association

**Table 4:** Results from multiple logistic regression analysis with Odds Ratios (OR) of factors affecting worsened outcomes in the two groups HFpEF and HFrEF

	Worsened			
	HFpEF ≥50%		HFrEF < 40%	
	OR	*p*-value	OR	*p*-value
Sex (female vs. male)			0.824	0.024
Clinic (cardiology vs. medicine/geriatric)			0.697	< 0.001
Fatigue (pronounced/severe limitations vs. light)			0.650	< 0.001
Shortness of breath (pronounced/severe limitations vs. light)			0.743	0.008
Atrial fibrillation/flutter (yes/no)	1.466	0.009		
Pulmonary disease (yes/no)			1.326	0.009
Reduced renal function (yes/no)	1.447	0.012		
MRA (yes/no)			0.820	0.019
Diuretic (yes/no)	0.526	< 0.001		

All analyses controlled for: Sex (female vs. male), Age (over 75 years old), Smoker (yes/no), Clinic (Cardiology vs. Medicine/Geriatrics), Follow up referral to hospital (Hospital vs. Primary care) Follow up referral to out-patient HF nurse (yes/no), NYHA classification grades III/IV vs. 1/II, Fatigue – pronounced/severe limitations, Shortness of breath – pronounced/severe limitations, Duration of heart failure > 6 months), Ischaemic heart disease, Previous myocardial infarction, Hypertension, Atrial fibrillation/flutter, Diabetes, Pulmonary disease, Anaemia, Mean heart rate bpm > 70, Renal function (e-GFR < 60 μmol/L), ACEI/ARB, ß-blocker, MRA, Diuretic

*Abbreviations*: *ACEI* angiotensin converting enzyme inhibitor, *ARB* angiotensin II receptor blocker, *EF* ejection fraction, *e-GFR* estimated glomerular filtration, *HFrEF* Heart Failure with Reduced Ejection Fraction, *HFpEF* Heart Failure with Preserved Ejection Fraction, *MRA* mineralocorticoid receptor antagonist, *NYHA* New York Heart Association

## Discussion

Our aim was to evaluate the usefulness of EQ-5D as a PROM analysing the specific dimensions. Especially, we used the Paretian classification of Health Change among two groups of patients with different clinical pictures of HF (HFpEF and HFrEF). While changes in the EQ-5D index showed small differences between the two HF groups over one year, we identified larger variations over time by analysing the data for each dimension of the EQ-5D. This also showed different patterns between the two groups, in which the HFpEF group showed higher proportions reporting problems in all dimensions, while in the HFrEF, it was found in only three dimensions (mobility, self-care, and pain/discomfort) with lower proportions in one (usual activities).

These findings are in line with the known clinical picture, where HFrEF patients do have a better clinical outcome and where ability to perform usual activities often is one of the first signs. The difference between the groups were further illuminated with help of the Paretian classification, by which we could, within each HF subgroup, identify those who had an overall improved HRQoL and those who had an overall worsening, as well as those with mixed changes and those with no changes. Again, patterns differed between the groups. Patients in the HFpEF group showed a larger proportion with overall worsening, and there was a smaller proportion showing overall improvements than those in the HFrEF group. This was independent of whether the group “no problems” was included or not. These differences and patterns would not have been discovered if we had used the EQ-5D index, and this kind of analysing the specific dimensions of EQ-5D can be valuable for the identification of patient groups with different needs regarding interventions.

Indeed, in a logistic regression one main difference between the patient groups on factors that indicated a worsened or an improved outcome was being treated at a cardiology clinic, which was significant only for patients with HFrEF [19]. Other important predictors for improved outcome in the HFrEF group were high fatigue, good renal function and little history of IHD or pulmonary disease at baseline. Predictors for overall worsening were male gender, low fatigue, low problems with breathing, prevalent pulmonary disease and low use of MRA at baseline. For the HFpEF group, factors related to improved outcome were severe problems with breathing and good renal function, while poor renal function, low use of diuretics and atrial fibrillation at baseline predicted poorer outcome.

These findings are supported by clinical experience and by earlier studies, where e.g. poor renal function as well as atrial fibrillation among patients with HF is associated with a poorer prognosis. Also, the patients in the HFpEF group consisted of mainly elderly women with more coexisting conditions who were usually treated according to less specific guidelines, and were often followed up in primary care; while patients in the HFrEF group are younger men with a history of IHD and previous myocardial infarction [20–22] who were followed up in hospital and treated according to established guidelines and many of these receive triple treatment with ACEI:s/ARB:s, beta-blockers and MRA:s all shown to improve the prognosis in patients with HFrEF [22, 23].

Over time you may expect a gradual worsening of HF, which in this study was more pronounced in the HFpEF patients than in the HFrEF patients. This may be explained by the fact that patients with HFrEF often have been given better treatment (according to existing guidelines) and therefore have less worsening HF and fewer symptoms over time. In patients with HFpEF we do not have any evidence-based treatment and therefore these patients are recommended to be treated based on their concomitant diseases resulting in more worsening HF and more symptoms over time. Based on this we are in urgent need of more treatment studies in this patient group. This is important, as previous studies have shown that the medical prognosis is similar in the two groups of patients (HFrEF and HFpEF) with poor survival and high rates of hospitalization [21], and this has now been corroborated by our findings of poorer HRQoL in the HFpEF group.

Patients with HFrEF had a shorter duration of HF and were more likely to be assessed as NYHA class III-IV than HFpEF patients, who were more often in NYHA class I-II and had had HF for a longer time period. In contrast to the NYHA classification, a higher proportion of patients in the HFpEF group reported moderate or high problems for all scales of EQ-5D and also reported more symptoms of fatigue and shortness of breath as severe limitations than those in the HFrEF group. This confirms the results of Ekman et al., who pointed out that patients’ self-assessed symptoms and their NYHA classification did not necessarily agree [24, 25]. This divergence between the results of medical examinations and those of patients’ self-assessed symptoms, (which has also been shown in other clinical conditions including chronic pulmonary disease) adds weight to the importance of the assessment of PROM [26].

Our results suggest that the method used for analysing EQ-5D is important. We found only minor differences over time and between groups in the EQ-5D index, so this measure does not give enough information about patients with HF. This is an illustration of the danger of using an index for composite measures in complex patient groups. Instead, we found notable and informative differences between the groups in levels of, and changes in specific dimensions.

Especially, our results illustrate the important feature of the Paretian classification of Health Change, in giving an instrument to help identify subgroups of patients with overall improvement or worsening and thus different needs for interventions in health services. The Paretian classification of Health Change is still relatively unknown but has been used, and been helpful, for analyses of different groups of patients before and after surgery [14].

### Limitations

We acknowledge the limitations of our study. The population was defined as those who had responded to EQ-5D at baseline, and of these patients 2853 (30%) were excluded because they had had no EQ-5D at follow up. However, this was mainly drop out for clinics and not for individual patients. One explanation for this drop out was that it is not part of the clinical routine to assess HRQoL in patients with HF. However, the characteristics of patients of our study were representative of HF patients in general according to a number of studies [9], and due to the aim of the study this will probably not affect the results. Moreover, as this is an observational, registry-based study, the population was unselected (in contrast to patients in randomised controlled studies) and therefore represents patients in the real clinical setting. However, our study design is also, as other registry-based studies, at risk of being subject to selection bias and confounding variables and due to this it is impossible to draw any conclusions of the different size of the OR:s for the variables, which are significant in our logistic regression analysis, and their clinical importance. Although our registry contains a large number of unique patients with extensive baseline variables, we cannot rule out inaccuracies in recorded data or unmeasured confounding variables. Several other questions remain concerning methods for analysing HRQoL among patients with HF, such as the optimal time for baseline assessments.

## Conclusion

Our results, comparing groups with HFpEF and HFrEF over one year showed that the usefulness of EQ-5D is dependent on the method used. The EQ-5D index showed minor differences between the two groups, while analyses of specific dimensions showed different patterns of change in the two groups. Especially, the Paretian classification identified subgroups that were characterised by overall improvement or worsening, and multiple linear regression models showed different determinants of improvement or worsening in these groups. This method can therefore be helpful in the identification of needs for improvement in health services.

## Data Availability

Data available on request due to privacy/ethical restrictions.
